# Risk prediction models for mortality in patients with severe pneumonia: a systematic review and meta-analysis

**DOI:** 10.3389/fmed.2025.1564545

**Published:** 2025-07-23

**Authors:** Xiaoyu Wang, Zhenzhen Feng, Lu Wang, Wenrui Liu, Jiansheng Li

**Affiliations:** ^1^Department of Respiratory Diseases, The First Affiliated Hospital of Henan University of Chinese Medicine, Zhengzhou, Henan, China; ^2^The First Clinical Medical College, Henan University of Chinese Medicine, Zhengzhou, Henan, China; ^3^Collaborative Innovation Center for Chinese Medicine and Respiratory Diseases Co-constructed by Henan Province & Education Ministry of P.R. China/Henan Key Laboratory of Chinese Medicine for Respiratory Diseases, Henan University of Chinese Medicine, Zhengzhou, Henan, China

**Keywords:** severe pneumonia, mortality, prediction model, systematic review, meta-analysis

## Abstract

**Background:**

The number of risk prediction models for mortality in patients with severe pneumonia (SP) is increasing, while the quality and clinical applicability of these models remain unclear. This study aimed to systematically review published research on risk prediction models for mortality in patients with SP.

**Methods:**

PubMed, Embase, Cochrane Library, and Web of Science were searched from inception to August 31, 2024. Data from selected studies were extracted, including study design, participants, diagnostic criteria, sample size, predictors, model development, and performance. The prediction model risk of bias assessment tool was used to assess the risk of bias and applicability. A meta-analysis of the area under the curve (AUC) values from validated models was conducted using Stata 17.0 software.

**Results:**

A total of 22 prediction models from 18 studies were included in this review, including 15 logistic regression models, two cox proportional regression hazards models, two classification and regression trees, one light gradient boosting machine, and one multilayer perceptron. The reported AUC values ranged from 0.713 to 0.952. Seventeen studies were found to have a high risk of bias, primarily due to inappropriate data sources and poor reporting of the analysis domain. The pooled AUC value of five validated models was 0.85 (95% confidence interval: 0.81–0.88), indicating a fair level of discrimination.

**Conclusion:**

Although the included studies reported that the risk prediction models for mortality in patients with SP exhibited a certain level of discriminative ability, most of these models were found to have a high risk of bias. Future studies should focus on developing new models with larger sample sizes, rigorous study designs, and multicenter external validation.

**Systematic review registration:**

https://www.crd.york.ac.uk/PROSPERO/view/CRD42024589877, identifier: CRD42024589877.

## 1 Introduction

Severe pneumonia (SP) is a common and serious disease characterized by lower respiratory infection with rapid progression, poor prognosis, and heavy economic burden. It is the leading cause of infection-related mortality and admissions to intensive care units (ICUs) globally (1). The Global Burden of Disease Study from 2016 states that the mortality of severe community-acquired pneumonia (SCAP) can range from 20% to 50% (2). Despite quick advancements in pertinent diagnosis and therapy, the mortality of SP has hardly dropped in recent years (1). According to a prospective cohort study conducted in the United States (US) in 2020, 23% of pneumonia patients needed to be admitted to ICUs. Pneumonia patients in ICUs had a 30-day mortality of 27% and an annual mortality of 47% (3).

Risk prediction models for mortality in patients with SP contribute to identifying high-risk patients with poor prognosis and intervening timely, which is significant for improving clinical outcomes. The Confusion, Urea, Respiratory rate, Blood pressure, age ≥ 65 (CURB-65) score is one of the commonly used pneumonia-related scoring systems in clinical practice. This simple scoring system rapidly stratifies patients into three distinct risk classes based on five key clinical parameters (4). The Pneumonia Severity Index (PSI) consists of 20 variables covering demographics, comorbidities, physical examination, and laboratory tests that can categorize patients into five risk classes, providing a more comprehensive assessment (5). The Sequential Organ Failure Assessment (SOFA) and the Acute Physiology and Chronic Health Evaluation II (APACHE II) provide a comprehensive assessment of organ dysfunction in the ICU (6, 7). The Predisposition, Insult, Response, Organ dysfunction (PIRO) score can be helpful in evaluating mortality in sepsis-associated pneumonia (8). However, they face some limitations. The CURB-65 score demonstrates suboptimal performance in critical patients and omits crucial inflammatory biomarkers. PSI suffers from practical constraints in emergency settings due to its complexity. SOFA and APACHE II lack pneumonia-specific parameters. The PIRO score shows restricted applicability in non-septic cases. Therefore, the development of specific prediction models for SP carries significant clinical implications.

This study aims to screen and systematically review existing risk prediction models for mortality in patients with SP. The findings will inform clinical decision-making and guide future research directions in this critical area.

## 2 Methods

This study was reported following the Preferred Reporting Items for Systematic Reviews and Meta-Analyses (PRISMA) (9) guidelines and the Critical Appraisal and Data Extraction for Systematic Reviews of Prediction Modeling Studies (CHARMS) (10) checklist. The protocol has been registered in the International Prospective Register of Systematic Reviews (PROSPERO) and the registry number is CRD42024589877.

### 2.1 Search strategy

We systematically searched the PubMed, Embase, Cochrane Library, and Web of Science databases without language restrictions from their inception to August 31, 2024. We used a combination of the following keywords to build the search strategy: (“severe”) AND (“pneumonia” OR “pulmonary inflammation” OR “pulmonary infection”) AND (“predict model” OR “risk prediction” OR (“risk score” OR “prediction model” OR “prognostic model” OR “risk factor” OR “nomogram” OR “machine learning” OR “deep learning” OR “artificial intelligence” OR “neural network” OR “decision tree” OR “computational intelligence” OR “machine intelligence” OR “bayesian” OR “k-nearest neighbor” OR “decision support” OR “random forest” OR “support vector machine” OR “Xgboost” OR “adaboost” OR “gradient boosting machine” OR “regression tree” OR “least squares” OR “stepwise regression” OR “linear model” OR “logistic regression” OR “principle component analysis” OR “independent component analysis” OR “k means clustering”). The detailed search strategy is provided in Supplementary material. We also identified additional relevant studies by reviewing the reference lists of the retrieved studies and review articles.

For the systematic review, we utilized the PICOTS system, recommended by CHARMS checklist (10). This system helps frame the review's aim, search strategy, and study inclusion and exclusion criteria. The key items of our systematic review are described below:

P (Population): Patients with severe pneumonia, as defined by either the guidelines published in 2007/2019 for CAP by the Infectious Diseases Society of America/American Thoracic Society (11, 12) or the Guidelines for the Diagnosis and Treatment of Adult Community-Acquired Pneumonia in China (2016 Edition) (13).

I (Intervention model): Risk prediction models for mortality in patients with SP that were developed and published (predictors ≥ 2).

C (Comparator): No competing model.

O (Outcome): The outcome focused on death.

T (Timing): The outcome was predicted after evaluating basic information at admission, clinical scoring scale results, and laboratory indicators.

S (Setting): The intended use of the risk prediction model is to individualize the prediction of mortality in patients with SP, facilitating the implementation of preventive measures to prevent adverse events.

### 2.2 Inclusion and exclusion criteria

The inclusion criteria for studies were: (1) studies involving patients aged ≥ 18 years with SP; (2) an observational study design; (3) reported a prediction model; (4) the outcome of interest was death.

The exclusion criteria for studies were: (1) studies that did not develop a prediction model; (2) only one predictor; (3) duplicate publications, reviews, editorials, animal studies, case reports, or other non-data driven article-types; (4) the full text could not be retrieved; (5) to enhance homogeneity, we excluded studies that explicitly focused on fungal and viral pneumonia, such as severe H1N1, SARS, or COVID-19.

### 2.3 Study selection

The selection process of the studies was conducted independently by two investigators. Initially, duplicate studies were removed, then the remaining studies were assessed based on titles and abstracts to determine eligibility. Following the inclusion and exclusion criteria, full texts were reviewed, and the reference lists of all eligible studies were examined to identify any potentially relevant studies. In case of disagreements regarding study selection, a discussion involving three investigators was held to reach a consensus.

### 2.4 Data extraction

The data was extracted by two investigators independently according to CHARMS checklist (10), including the name of the first author, publication year, country, study design, participants, diagnostic criteria, sample size, model development method, variable selection method, model validation type, model performance measures, handling of missing data, method for processing continuous variables, final predictors used in the model, and the form in which the model was presented. In case of disagreements regarding data extraction, a discussion involving three investigators was held to reach a consensus.

### 2.5 Quality assessment

Two independent investigators used the prediction model risk of bias assessment tool (PROBAST) (14, 15) to evaluate the bias risk and applicability of the included studies. The evaluation of bias risk comprises 20 signaling questions categorized into four domains: participants, predictors, outcome, and analysis. Each signaling question can be answered as “yes,” “probably yes,” “no,” “probably no,” or “no information.” Each domain can be judged as “low risk of bias,”“high risk of bias,” or “unclear.” The evaluation of applicability comprises three domains: participants, predictors, and outcome.

### 2.6 Statistical analysis

Qualitative analysis method was used to sort out the general information and model information. A meta-analysis of the area under the curve (AUC) values from validated models was conducted using Stata software (version 17.0). A random-effects model was applied if there was significant heterogeneity; otherwise, a fixed-effects model was used. The Chi-square test and *I*^2^ value were implemented to assess heterogeneity, and *p* < 0.1 or *I*^2^ > 50% indicated significant heterogeneity. When statistical heterogeneity existed, we conducted sensitivity analyse to verify the robustness of the overall results, which were carried out by gradually removing studies. Egger's test was used to identify publication bias, with *p* > 0.05 indicating a low likelihood of publication bias.

## 3 Results

### 3.1 Study selection

**Figure 1:**
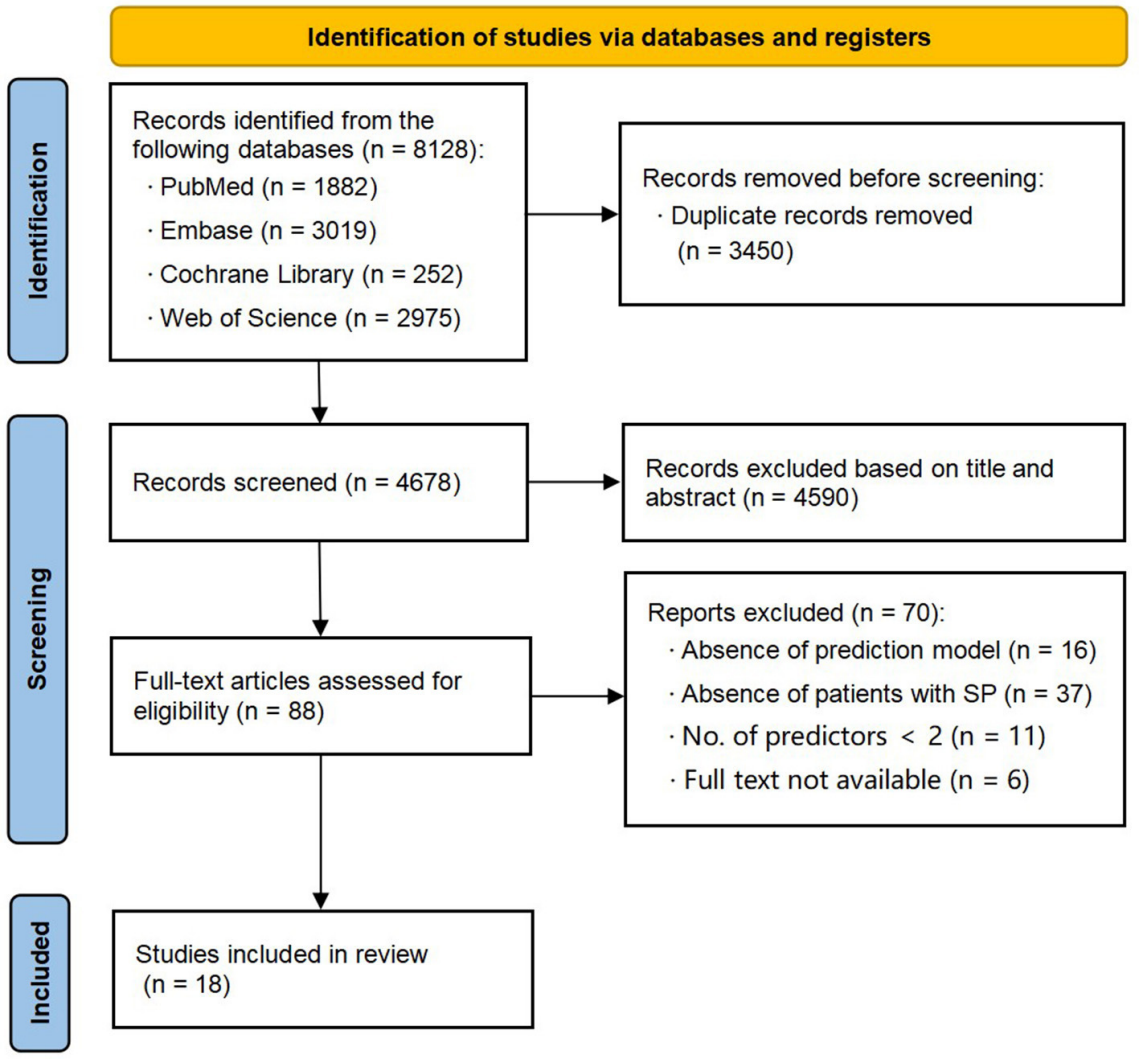
Preferred Reporting Items for Systematic reviews (PRISMA) flowchart of literature search and selection.

The initial search yielded a total of 8,128 records. After removing 3,450 duplicate records, 4,678 titles and abstracts were screened for eligibility. Following this screening process, 88 articles were included for further evaluation. During the subsequent evaluation, 37 studies were excluded as their participants did not meet the criteria. Additionally, 16 studies did not establish prediction models, 11 studies had only one predictor, and six studies were not available. Ultimately, we got 18 studies (16–33) that met all of the inclusion criteria. The selection procedure is illustrated in Figure 1.

### 3.2 Study characteristics

Out of 18 studies, 15 studies (18–29, 31–33) were conducted in China, with one study each from the US, Spain, and South Korea. In terms of study design, ten studies (19–21, 25, 26, 29–33) were retrospective cohort, six studies (16, 17, 22, 24, 27, 28) were prospective cohort, and two studies (18, 23) were case-control. The sample sizes ranged from 94 to 37,348 cases, and the patient mortality ranged from 21.55% to 55.32%. Detailed characteristics are presented in Table 1.

**Table 1:** Overview of basic data of the included studies.

**Author**	**Year**	**Country**	**Study design**	**Participants**	**Diagnostic criteria**	**Outcome cases/sample size (%)**	**Main outcome**
El-Solh et al. (16)	2001	US	Prospective cohort	≥75, SCAP	–	57/104 (54.81)	Hospital mortality
Sirvent et al. (17)	2013	Spain	Prospective cohort	SCAP	A	56/242 (23.14)	28-day mortality
Wang et al. (18)	2017	China	Case-control	SCAP	A	15,921/373,48 (42.63)	28-day mortality
Huang et al. (19)	2021	China	Retrospective cohort	SCAP with T2DM	A	444/1,262 (35.18)	Hospital mortality
Gong et al. (20)	2022	China	Retrospective cohort	≥65, SCAP with CVD	A	220/413 (53.27)	Hospital mortality
Huang et al. (21)	2022	China	Retrospective cohort	SCAP with COPD	A	361/873 (41.35)	Hospital mortality
Song et al. (22)	2022	China	Prospective cohort	≥65, SCAP	A	65/292 (22.27)	28-day mortality
Gao et al. (23)	2023	China	Case-control	SCAP	A	32/94 (34.04)	28-day mortality
Lu et al. (24)	2023	China	Prospective cohort	SCAP	A	40/158 (25.32)	Hospital mortality
Pan et al. (25)	2023	China	Retrospective cohort	SCAP	A	117/543 (21.55)	Hospital mortality
Wang et al. (26)	2023	China	Retrospective cohort	SAP	A/B	52/94 (55.32)	90-day mortality
Shang et al. (27)	2023	China	Prospective cohort	≥65, SCAP	A	177/490 (36.12)	Hospital mortality
Zhang et al. (28)	2023	China	Prospective cohort	SP	B	45/152 (29.61)	28-day mortality
Gao et al. (29)	2023	China	Retrospective cohort	SP with EN	A	225/632 (35.60)	Hospital mortality
Jeon et al. (30)	2023	Korea	Retrospective cohort	SP	A	223/816 (27.33)	ICU mortality
Miao et al. (31)	2024	China	Retrospective cohort	≥65, SCAP	A	88/406 (21.67)	28-day mortality
Wei et al. (32)	2024	China	Retrospective cohort	≥65, SCAP	A	868/2,365 (36.70)	Hospital mortality
Zhang et al. (33)	2024	China	Retrospective cohort	SCAP	A	–/815	30-day mortality

T2DM, Type 2 diabetes; CVD, cerebrovascular disease; COPD, chronic obstructive pulmonary disease; SAP, severe aspiration pneumonia; SP, severe pneumonia; EN, enteral nutrition; A, the guidelines published in 2007/2019 for CAP by the Infectious Diseases Society of America/American Thoracic Society; B, the Guidelines for the Diagnosis and Treatment of Adult Community-Acquired Pneumonia in China (2016 Edition); –, not reported.

### 3.3 Model construction

A total of 22 models were reported, including 15 logistic regression models, two cox proportional regression hazards models, two classification and regression trees (CART), one light gradient boosting machine (LightGBM), and one multilayer perceptron (MLP). Regarding the model development and validation processes, the studies demonstrated varying scopes. Four studies (17, 23, 26, 31) were limited to model development. Twelve studies (16, 18–22, 24, 27–30, 32) conducted both model development and internal validation. One study (25) conducted model development and external validation. Notably, only one study (33) comprehensively covered model development, internal validation, and external validation. The sample size for model development was 94–37,348 cases, while 40–710 cases were for model validation. The number of candidate variables considered during the model construction process varied from 12 to 59, with the final models retaining 2–16 predictors. Continuous variables were transformed into categorical variables based on clinically significant cutoff values or the upper and lower limits of the normal range in two studies (19, 20). The variable selection methods were also diverse. Sixteen studies (16–29, 32, 33) reported the variable selection methods, including univariable analysis, multivariable analysis, least absolute shrinkage and selection operator (LASSO), and recursive feature elimination (RFE). Seven studies (19, 21, 25, 27, 29, 30, 33) detailed the approaches to handling missing data, primarily through multiple imputation (MI) and case elimination. Detailed information of model construction is shown in Table 2.

**Table 2:** Overview of model construction of the included studies.

**Author**	**Model development method**	**Model type**	**Variable selection**	**Number of candidate variables**	**Continuous variable processing**	**Sample size (D/I/E)**	**Missing data handling**	**Final predictors**
El-Solh et al. (16)	CART, LR	D, I	Univariable analysis, RFE	19	Continuous	104/–/–	–	Use of vasopressor, multilobar pneumonia, BUN/Cr, GCS, UO, ADL
Sirvent et al. (17)	LR	D	Univariable and multivariable analysis	34	Continuous	242/–/–	–	Age, CURB score, septic shock, ARDS, ARF
Wang et al. (18)	CART	D, I	Multivariable analysis	31	Continuous	373,48/–/–	–	Cr, WBC, CRP, GCS, ${\text{HCO}}_{3}^{-}$
Huang et al. (19)	LR	D, I	Univariable and multivariable analysis	17	Categorical	883/379/–	MI/elimination	Number of comorbidities, diabetes related complications, BP, CRP, NLR, BNP, lactate
Gong et al. (20)	LR	D, I	Univariable and multivariable analysis, LASSO	34	Categorical	413/–/–	–	Age, use of vasopressor, number of primary symptoms, temperature, monocyte, CRP, NLR
Huang et al. (21)	LR	D, I	Univariable and multivariable analysis	17	Continuous	611/262/–	MI	Age, diabetes, CKD, SBP, fibrinogen, IL-6, BUN
Song et al. (22)	LR	D, I	Univariable and multivariable analysis	36	Continuous	292/–/–	–	Age, GCS, platelet, BUN
Gao et al. (23)	LR	D	Univariable and multivariable analysis	12	Continuous	94/–/–	–	MicroRNA-24, microRNA-233, APACHE II
Lu et al. (24)	LR	D, I	Univariable and multivariable analysis	18	Continuous	118/40/–	–	IL-6, BUN, PCT, length of stay, bacterial infection, mNGS
Pan et al. (25)	LR	D, E	Univariable and multivariable analysis	23	Continuous	455/–/88	MI/elimination	Lymphocyte, PaO_2_/FiO_2_, shock, APACHE II
Wang et al. (26)	Cox	D	Univariable and multivariable analysis	-	Continuous	94/–/–	–	Platelet, total protein
Shang et al. (27)	LR	D, I	Multivariable analysis, LASSO	59	Continuous	490/–/–	MI	MAP, SpO_2_, GCS, LDH, lactate, BUN, ESMcsa
Zhang et al. (28)	LR	D, I	Multivariable analysis	15	Continuous	152/–/–	–	APACHE II, NLR, lactate, CAR
Gao et al. (29)	LR	D, I	Univariable and multivariable analysis	28	Continuous	632/–/–	MI/elimination	Duration of mechanical ventilation, combined malignant proliferative disease, platelet, PT, ALT, albumin, K^+^, Na^+^
Jeon et al. (30)	LR, LightGBM, MLP	D, I	-	55	Continuous	489/327/–	MI/elimination	PaO_2_/FiO_2_, CRP, lactate, UO, SBP, WBC, troponin, PaO_2_, PT-INR, ALP, PR, PaCO_2_, pH, steroid, norepinephrine, DBP
Miao et al. (31)	Cox	D	-	-	Continuous	406/–/–	–	NLR, SHR
Wei et al. (32)	LR	D, I	Univariable and multivariable analysis	26	Continuous	1,655/710/–	–	Age, use of vasopressor, CKD, neutrophil, platelet, BUN
Zhang et al. (33)	LR	D, I, E	LASSO	44	Continuous	502/216/97	Elimination	Age, combined malignant tumor, heart rate, MAP, albumin, BUN, PT, lactate

LR, logistic regression model; Cox, cox proportional regression hazards model; D, development; I, internal validation; E, external validation; BUN, blood urea nitrogen; Cr, creatinine; GCS, glasgow coma scale; UO, urinary output; ADL, activities of daily living score; ARDS, acute respiratory distress syndrome; ARF, acute renal failure; WBC, white blood cell; CRP, C-reactive protein; BP, blood pressure; NLR, neutrophil to lymphocyte ratio; CKD, chronic kidney disease; SBP, systolic blood pressure; IL-6, interleukin 6; BNP, brain natriuretic peptide; APACHE II, acute physiology and chronic health evaluation II; PCT, procalcitonin; mNGS, metagenomic next generation sequencing; MAP, mean arterial pressure; SpO_2_, blood oxygen saturation; LDH, lactate dehydrogenase; ESMcsa, cross-sectional area of erector spinae muscle; CAR, C-reactive protein to albumin ratio; PT, prothrombin time; ALT, alanine aminotransferase; SHR, stress hyperglycemia ratio; PaO_2_, partial pressure of oxygen; PT-INR, prothrombin time international normalized ratio; ALP, alkaline phosphatase; PR, pulse rate; PaCO_2_, partial pressure of carbon dioxide; pH, power of hydrogen; DBP, diastolic blood pressure.

### 3.4 Model performance

All the included studies reported AUC of model development, with values ranging from 0.713 to 0.952. Nine studies (19–21, 24, 27, 30, 32, 33) reported AUC values of internal validation, spanning 0.728 to 0.921, while two studies (25, 33) reported AUC values of external validation ranging from 0.778 to 0.893. Thirteen studies (17, 19–22, 24, 25, 27–30, 32, 33) evaluated model calibration using a calibration curve, Hosmer-Lemeshow test, or Brier score. Five studies (16, 23, 26, 28, 31) reported model specificity ranging from 69.05% to 93.30% and sensitivity ranging from 76.90% to 96.90%. The model accuracy was reported in just one study (26) at 80.85%. Eleven studies (19–22, 25, 27–30, 32, 33) evaluated clinical applicability using decision curve analysis (DCA). The results consistently demonstrated that the models provided substantial net benefits across a wide range of threshold probabilities, indicating robust clinical applicability. Notably, Huang et al. (21) further plotted a clinical impact curve (CIC) to predict improved probability stratification for a population size of 1,000. To further investigate the clinical utility of the prediction model, they established clinically meaningful cutoffs by categorizing nomogram scores into three risk strata: < 150 (low risk), 150–200 (moderate risk), and >200 (high risk) points. This stratification demonstrated remarkable risk discrimination. Thirteen studies (16, 18–22, 24, 27–30, 32, 33) reported internal validation methods, comprising seven using bootstrap resampling, one with 5-fold cross validation, three with 10-fold cross validation, and two utilizing random split validation. Notably, only two studies (25, 33) performed external validation, both implementing spatial validation through geographically distinct cohorts. The models were presented by nomogram in 11 studies (19–22, 24, 25, 27–29, 32, 33), decision tree in two studies (16, 18), and β coefficient of each factor in one study (23). Detailed information of model performance is shown in Table 3.

**Table 3:** Overview of model performance of the included studies.

**Author**	**AUC(D/I/E)**	**Calibration method**	**A/B/C(%)**	**Clinical applicability**	**Validation method**	**Model presentation**	**AUC of commonly used scoring systems**
El–Solh et al. (16)	Model 1: 0.932/–/– Model 2: 0.801/–/–	–	Model 1: 93.30/83.80/– Model 2: 83.30/76.90/–	–	Internal validation (5–FCV)	Decision tree	APACHE II (0.711)
Sirvent et al. (17)	0.863/–/–	Hosmer–Lemeshow test	–	–	–	–	
Wang et al. (18)	0.889/–/–	–	–	–	Internal validation (10–FCV)	Decision tree	APACHE II (0.864), SOFA (0.877), PSI (0.761), CURB−65 (0.767)
Huang et al. (19)	0.907/0.873/–	Calibration curve	–	DCA	Internal validation (bootstrap)	Nomogram	PSI (0.809)
Gong et al. (20)	0.800/0.781/–	Calibration curve	–	DCA	Internal validation (bootstrap)	Nomogram	PSI (0.624), CURB−65 (0.630)
Huang et al. (21)	0.840/0.830/–	Calibration curve	–	DCA/CIC	Internal validation (random split validation)	Nomogram	
Song et al. (22)	0.713/–/–	Calibration curve	–	DCA	Internal validation (bootstrap)	Nomogram	APACHE II (0.628), SOFA (0.660), PSI (0.576)
Gao et al. (23)	0.952/–/–	–	77.40/96.90/–	–	–	β coefficient of each factor	APACHE II (0.791)
Lu et al. (24)	0.829/0.921/–	Calibration curve	–	–	Internal validation (bootstrap)	Nomogram	
Pan et al. (25)	0.850/–/0.893	Calibration curve	–	DCA	External validation (spatial validation)	Nomogram	APACHE II (0.795/–/0.746), SOFA (0.690/–/0.742)
Wang et al. (26)	0.832/–/–	–	69.05/90.38/80.85	–	–	–	
Shang et al. (27)	Model 1: 0.803/–/– Model 2: 0.836/0.826/–	Calibration curve, Hosmer–Lemeshow test, Brier score	–	DCA	Internal validation (bootstrap)	Nomogram	APACHE II (0.740), SOFA (0.778), PSI (0.704), CURB−65 (0.669)
Zhang et al. (28)	0.826/0.848/–	Calibration curve	87.32/88.45/–	DCA	Internal validation (bootstrap)	Nomogram	
Gao et al. (29)	0.782/–/–	Calibration curve, Hosmer–Lemeshow test	–	DCA/CIC	Internal validation (bootstrap)	Nomogram	
Jeon et al. (30)	Model 1: 0.820/0.832/– Model 2: 0.827/0.840/– Model 3: 0.838/0.856/–	Calibration curve, Brier score	–	DCA	Internal validation (10–FCV)	–	APACHE II (0.616), SOFA (0.619), SAPS II (0.650)
Miao et al. (31)	0.898/–/–	–	81.10/89.80/–	–	–	–	
Wei et al. (32)	0.742/0.728/–	Calibration curve	–	DCA	Internal validation (random split validation)	Nomogram	
Zhang et al. (33)	0.803/0.756/0.778	Calibration curve, Hosmer–Lemeshow test	–	DCA	Internal validation (10–FCV), external validation (spatial validation)	Nomogram	

A, specificity; B, sensitivity; C, accuracy; DCA, decision curve analysis; CIC, clinical impact curve; 5-FCV, 5-fold cross validation; 10-FCV, 10-fold cross validation; APACHE II, acute physiology and chronic health evaluation II; SOFA, sequential organ failure assessment; CURB-65, confusion, urea, respiratory rate, blood pressure, age ≥ 65; PSI, pneumonia severity index; SAPS II, simplified acute physiology score II.

### 3.5 Quality assessment

#### 3.5.1 Risk of bias assessment

In the participants domain, 12 studies (18–21, 23, 25, 26, 29–33) were judged as “high risk of bias” due to retrospective study design. In the predictors domain, two studies (23, 26) were classified as “high risk of bias” due to the inclusion of statistically nonsignificant predictors, while one study (30) was rated as “unclear.” In the outcome domain, all the included studies were assessed as “low risk of bias.” In the analysis domain, 17 studies (16–26, 28–33) were categorized as “high risk of bias.” The reasons were as follows. Events per variable (EPV) were fewer than 10 in 10 studies (16, 17, 22–25, 28–30, 33) during model development. Model validation sample sizes were below 100 in two studies (24, 25). Two studies (19, 20) transformed continuous variables into categorical variables. One study (33) eliminated participants with missing data. Five studies (16, 18, 23, 26, 31) failed to evaluate model calibration altogether. One study (17) relied solely on the Hosmer-Lemeshow test for calibration assessment. Internal validation was absent in five studies (17, 23, 25, 26, 31), and two studies (21, 32) only used random split verification. The results of risk of bias assessment are shown in Table 4.

**Table 4:** Overview of quality assessment of the included studies.

**Author**	**Risk of bias**				**Applicability**			**Overall**	
	**Participants**	**Predictors**	**Outcome**	**Analysis**	**Participants**	**Predictors**	**Outcome**	**Risk of bias**	**Applicability**
El–Solh et al. (16)	+	+	+	–	+	+	+	–	+
Sirvent et al. (17)	+	+	+	–	+	+	+	–	+
Wang et al. (18)	–	+	+	–	+	+	+	–	+
Huang et al. (19)	–	+	+	–	+	+	+	–	+
Gong et al. (20)	–	+	+	–	+	+	+	–	+
Huang et al. (21)	–	+	+	–	+	+	+	–	+
Song et al. (22)	+	+	+	–	+	+	+	–	+
Gao et al. (23)	–	–	+	–	+	+	+	–	+
Lu et al. (24)	+	+	+	–	+	+	+	–	+
Pan et al. (25)	–	+	+	–	+	+	+	–	+
Wang et al. (26)	–	–	+	–	+	+	+	–	+
Shang et al. (27)	+	+	+	+	+	+	+	+	+
Zhang et al. (28)	+	+	+	–	+	+	+	–	+
Gao et al. (29)	–	+	+	–	+	+	+	–	+
Jeon et al. (30)	–	?	+	–	+	+	+	–	+
Miao et al. (31)	–	+	+	–	+	+	+	–	+
Wei et al. (32)	–	+	+	–	+	+	+	–	+
Zhang et al. (33)	–	+	+	–	+	+	+	–	+

–, high risk of bias; +, low risk of bias; ?, unclear.

#### 3.5.2 Applicability accessment

The included studies have shown good applicability in different domains, as shown in Table 4.

### 3.6 Meta-analysis

**Figure 2:**
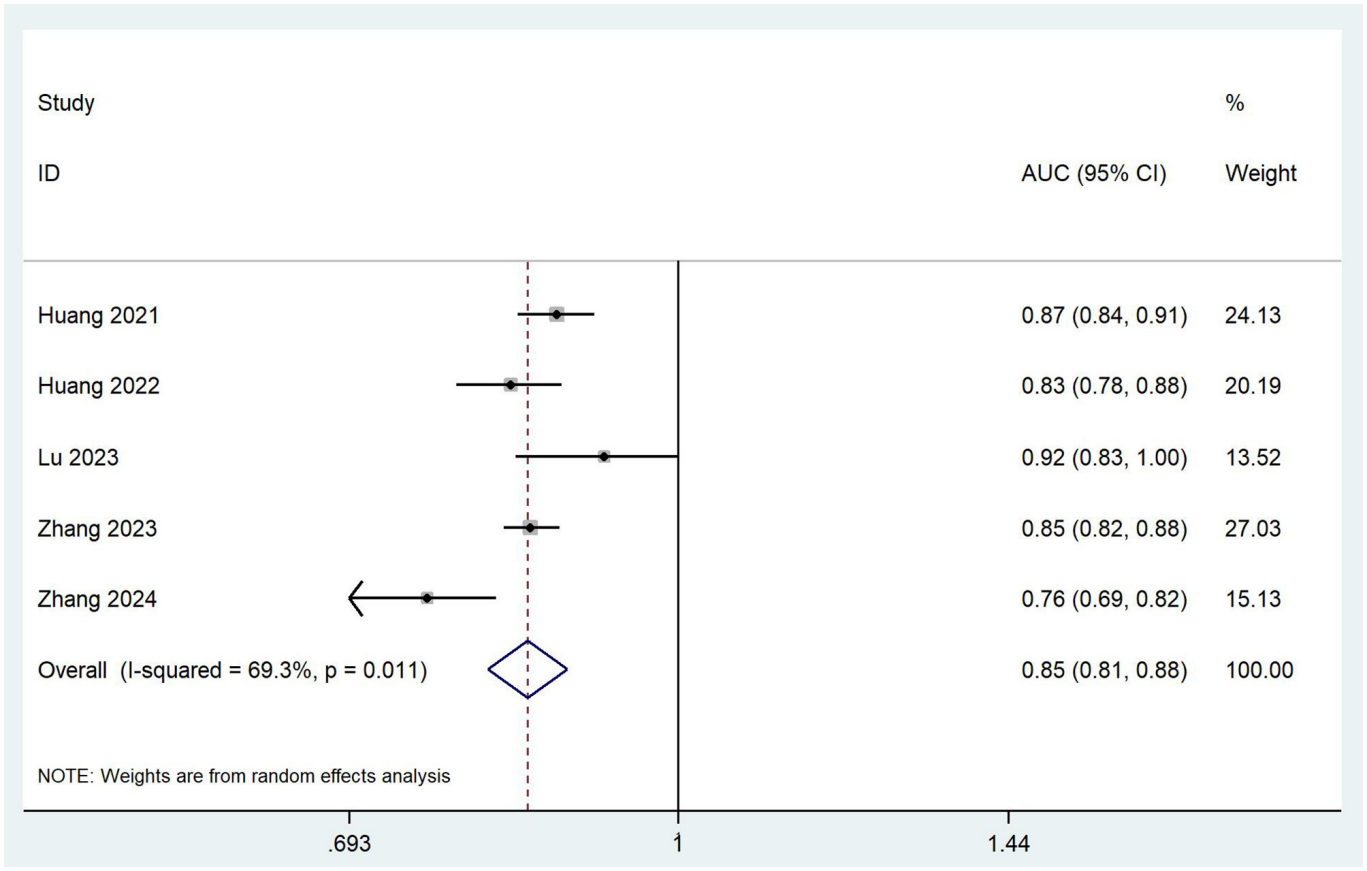
Forest plot of the random effects meta-analysis of pooled AUC estimates for five validation models.

To ensure the accuracy and reliability of model performance evaluation, only AUC values from validated models were included in the meta-analysis. This selective approach aligns with CHARMS guideline recommendations, which emphasize prioritizing models with complete validation data when synthesizing prediction model performance in meta-analyses. Owing to insufficient reporting on the validation details in most included studies, only five studies (19, 21, 24, 28, 33) that provided complete information on the validation AUC values and 95% confidence intervals (CI) qualified for the meta-analysis. The *I*^2^ value was 69.3% (*p* = 0.011), indicating significant heterogeneity among the studies. The pooled AUC was calculated using a random-effects model, resulting in a value of 0.85 (95% CI: 0.81–0.88), as shown in Figure 2. Sensitivity analysis confirmed the robustness of the result. The regression value from Egger's test (p = 0.764) indicated no significant publication bias.

## 4 Discussion

We evaluated 22 models that demonstrated moderate to good predictive performance, with reported AUC ranging from 0.713 to 0.952. The pooled AUC value of five validated models included in the meta-analysis was 0.85 (95% CI: 0.81–0.88), indicating strong discriminative ability. Several included studies compared the models with commonly used scoring systems. The results indicated that the newly developed models exhibited superior discriminative ability, although this may be attributed to the specificity of patients. DCA and CIC further supported the utility of these models, demonstrating a favorable net benefit across a wide range of threshold probabilities, which underscores their potential for clinical decision-making. Evidently, beyond their robust predictive performance, these models provide significant clinical value in real clinical settings. First, prediction models facilitate early identification of high-risk patients, allowing clinicians to prioritize critical care resources and interventions. Second, these models assist in shared decision-making between clinicians and patients. By providing validated, objective mortality risk estimates, patients and families can better understand prognosis and make informed choices about treatment intensity. Third, they can guide the optimization of treatment strategies. By analyzing the variables associated with mortality in the models, clinicians can focus on modifying modifiable risk factors, such as time-to-antibiotic administration. Additionally, they can help in evaluating the effectiveness of new treatment modalities. By comparing observed mortality rates with predicted rates in patients receiving novel therapies, researchers and clinicians can assess whether the new interventions are having a positive impact on patient outcomes. To maximize clinical impact, Future research must bridge specificity and generalizability by developing both subtype-targeted models and flexible frameworks for complex clinical scenarios.

Notably, El-Solh et al. and Jeon et al. utilized both logistic regression and machine learning (ML) methods during model development, with the latter yielding better performance. One study (34) indicated that ML tend to yield higher accuracy compared to traditional logistic regression. With the development of artificial intelligence (AI) in medical domain, ML have shown various advantages (35). First, AI and ML algorithms can handle complex, high-dimensional data more effectively than traditional models. Patients with SP often present with a vast array of clinical, laboratory, and imaging data. AI/ML methods can uncover hidden patterns and non-linear relationships within these data, leading to more precise prediction models. Second, these advanced techniques can adapt and improve continuously. AI/ML models can be retrained to incorporate new information and refine prediction algorithms. This is a critical advantage in the context of evolving antimicrobial resistance patterns and new pathogens. Third, AI/ML methods can potentially provide personalized risk assessment. By analyzing individual patient characteristics, including genetic profiles, comorbidities, and disease progression trajectories, these models can generate more individualized mortality risk estimates, enabling more targeted and effective clinical decision-making. While the current reviews focused on traditional logistic regression models, AI/ML are likely to play an increasingly important role in improving the accuracy of mortality prediction in patients with SP, by leveraging their unique capabilities in data analysis, adaptability, and personalization.

Seventeen studies were assessed as having a high risk of bias, significantly limiting the practical utility of their prediction models. Participants and analysis domains were the primary sources of bias risk. Twelve retrospective studies faced risks of data missing, incomplete predictor inclusion, and inconsistent measuring methods. In contrast, prospective study designs could effectively mitigate these methodological shortcomings and substantially reduce the risk of bias. According to the PROBAST guidelines, an events-per-variable (EPV) ratio of at least 10 is recommended during model development to prevent overfitting. More candidate predictors and insufficient sample size in studies resulted in high risk of bias. The categorization of continuous variables leads to a loss of statistical information, while excluding participants with missing data similarly reduces statistical power. For a comprehensive assessment of model performance, calibration should be evaluated through more robust metrics such as calibration curves and the Brier score, rather than relying solely on the Hosmer-Lemeshow test. Additionally, most models lacked external validation, a key step to evaluate the generalization ability of the models. Future research should refer to PROBAST and transparent reporting of a multivariable prediction model for individual prognosis or diagnosis (TRIPOD) (36) for study design and reporting, prioritizing prospective approaches, ensuring adequate EPV and sample size, employing appropriate missing data handling, and conducting rigorous internal and external validation to reduce the risk of bias.

The included models contained 2–16 predictors, with the most frequently identified variables including age, APACHE II, glasgow coma scale (GCS), blood urea nitrogen (BUN), C-reactive protein (CRP), neutrophil to lymphocyte ratio (NLR), platelet, lactate, and use of vasopressor. With advancing age, the body's immune defense gradually weakens, leading to higher mortality in elderly patients with SP, particularly among the very aged (37). APACHE II has been widely adopted in clinical evaluations of critical diseases, remaining the global gold standard for prognostic evaluation in the ICU (38). As a standardized measure of consciousness levels, GCS has been demonstrated to be independently associated with the prognosis in CAP patients requiring ICU admission (39). A recent meta-analysis (40) confirmed BUN as an independent predictor of prognosis in patients with SP. Thrombocytopenia is prevalent in critically ill patients, often serving as an indicator of severe organ dysfunction and the development of intravascular coagulation (41). Elevated CRP and NLR levels reflect a sustained systemic inflammatory response in patients. The inflammatory storm triggers the production of various inflammatory factors, which can cause systemic immune damage in patients with SP. Studies have shown that both CRP and NLR are independently associated with occurrence and prognosis of critical disease (42, 43). Lactate has shown independent prognostic value in patients with critical diseases, particularly sepsis. Furthermore, the fluid resuscitation guided by lactate monitoring can improve patient outcomes (44). Vasopressor can be used as a combination pressor therapy in patients with refractory septic shock when catecholamines alone are ineffective, but there is a risk of visceral ischaemia (45). The predictors included in the 22 models may serve as potential predictors for future model development and inform subsequent investigations into critical risk factor analysis.

### 4.1 Limitations

The review has several potential limitations. Firstly, most of the included studies were conducted in China, which limits the applicability of the findings to other countries. Thus, it is important for future research to develop risk prediction models for mortality in patients with SP in diverse populations. Secondly, due to the differences in reporting transparency and methods of the included studies, our meta-analysis only integrated the AUC values of five validated models. A wealth of information in models could not be quantitatively analyzed. However, these issues did not affect the assessment of models and reflect methodological and reporting issues that exist in studies. More rigorous methodologies and more transparent reporting are needed in the future.

### 4.2 Conclusion

This systematic review conducted a descriptive analysis of 18 studies with 22 models and a meta-analysis of five validated models, indicating a certain level of discrimination. However, 17 studies were assessed as having a high risk of bias according to PROBAST. Therefore, researchers need to familiarize themselves with the PROBAST checklist and comply with the reporting guidelines outlined in the TRIPOD statement to improve the quality of future studies. Future research should combine ML to prioritize the development of new models with larger sample sizes, rigorous study designs, and multicenter external validation. In addition, researchers should translate models into a web calculator or application, and make risk classification, so that medical staff can implement targeted hierarchical prevention and management strategies. Making the prediction models more intelligent and convenient to better serve the clinic is also the focus of future research.

## Data Availability

The original contributions presented in the study are included in the article/Supplementary material, further inquiries can be directed to the corresponding author.
