# Supplementary material for: Risk prediction models for mortality in patients with severe pneumonia: a systematic review and meta-analysis
Source: Front Med (Lausanne). 2025 Jul 23;12:1564545. doi: 10.3389/fmed.2025.1564545 (PMC12325059; doi:10.3389/fmed.2025.1564545)
Supplement: Supplementary file 1 [file Table_1.docx]

Supplementary Material

# Search Strategys

Search strategies for the different databases ran on August 31, 2024.

PubMed (1882)

| Search | Query |
| --- | --- |
| #1 | "severe"[Title/Abstract] |
| #2 | (("pneumonia"[Title/Abstract]) OR ("pulmonary inflammation"[Title/Abstract])) OR ("pulmonary infection"[Title/Abstract]) |
| #3 | (((((("predict model"[Title/Abstract]) OR ("risk prediction"[Title/Abstract])) OR ("risk score"[Title/Abstract])) OR ("prediction model"[Title/Abstract])) OR ("prognostic model"[Title/Abstract])) OR ("risk factor"[Title/Abstract])) OR ("nomogram"[Title/Abstract]) |
| #4 | (((((((((((((((((((((("machine learning"[Title/Abstract]) OR ("deep learning"[Title/Abstract])) OR ("artificial intelligence"[Title/Abstract])) OR ("neural network"[Title/Abstract])) OR ("decision tree"[Title/Abstract])) OR (" computational intelligence"[Title/Abstract])) OR ("machine intelligence"[Title/Abstract])) OR ("bayesian"[Title/Abstract])) OR ("k-nearest neighbour"[Title/Abstract])) OR ("decision support"[Title/Abstract])) OR ("random forest"[Title/Abstract])) OR ("support vector machine"[Title/Abstract])) OR ("Xgboost"[Title/Abstract])) OR ("adaboost"[Title/Abstract])) OR ("gradient boosting machine"[Title/Abstract])) OR ("regression tree"[Title/Abstract])) OR ("least squares"[Title/Abstract])) OR ("stepwise regression"[Title/Abstract])) OR ("linear model"[Title/Abstract])) OR ("logistic regression"[Title/Abstract])) OR ("principle component analysis"[Title/Abstract])) OR ("independent component analysis"[Title/Abstract])) OR ("k means clustering"[Title/Abstract]) |
| #5 | #3 OR #4 |
| #6 | ("COVID-19"[Title/Abstract]) OR ("SARS-CoV-2" [Title/Abstract]) |
| #7 | #1 AND #2 AND #5 NOT #6 |

Embase (3019)

| Search | Query |
| --- | --- |
| #1 | 'severe':ti,ab,kw |
| #2 | (pneumonia:ti,ab,kw OR 'pulmonary inflammation':ti,ab,kw OR 'pulmonary infection':ti,ab,kw) NOT 'covid 19':ti,ab,kw NOT 'sars cov 2':ti,ab,kw |
| #3 | 'predict model':ti,ab,kw OR 'risk prediction':ti,ab,kw OR 'risk score':ti,ab,kw OR 'prediction model':ti,ab,kw OR 'prognostic model':ti,ab,kw OR 'risk factor':ti,ab,kw OR 'nomogram':ti,ab,kw |
| #4 | 'machine learning':ti,ab,kw OR 'deep learning':ti,ab,kw OR 'artificial intelligence':ti,ab,kw OR 'neural network':ti,ab,kw OR 'decision tree':ti,ab,kw OR 'computational intelligence':ti,ab,kw OR 'machine intelligence':ti,ab,kw OR bayesian:ti,ab,kw OR 'k-nearest neighbour':ti,ab,kw OR 'decision support':ti,ab,kw OR 'random forest':ti,ab,kw OR 'support vector machine':ti,ab,kw OR xgboost:ti,ab,kw OR adaboost:ti,ab,kw OR 'gradient boosting machine':ti,ab,kw OR 'regression tree':ti,ab,kw OR 'least squares':ti,ab,kw OR 'stepwise regression':ti,ab,kw OR 'linear model':ti,ab,kw OR 'logistic regression':ti,ab,kw OR 'principle component analysis':ti,ab,kw OR 'independent component analysis':ti,ab,kw OR 'k means clustering':ti,ab,kw |
| #5 | #3 OR #4 |
| #6 | #1 and #2 and #5 |

Cochrane Library (252)

| Search | Query |
| --- | --- |
| #1 | ("severe"):ti,ab,kw |
| #2 | ("pneumonia"):ti,ab,kw OR ("pulmonary inflammation"):ti,ab,kw OR ("pulmonary infection"):ti,ab,kw |
| #3 | ("predict model"):ti,ab,kw OR ("risk prediction"):ti,ab,kw OR ("risk score"):ti,ab,kw OR ("prediction model"):ti,ab,kw OR ("prognostic model"):ti,ab,kw OR ("risk factor"):ti,ab,kw OR ("nomogram"):ti,ab,kw |
| #4 | ("machine learning"):ti,ab,kw OR ("deep learning"):ti,ab,kw OR ("artificial intelligence"):ti,ab,kw OR ("neural network"):ti,ab,kw OR ("decision tree"):ti,ab,kw OR ("computational intelligence"):ti,ab,kw OR ("machine intelligence"):ti,ab,kw OR ("bayesian"):ti,ab,kw OR ("k-nearest neighbour"):ti,ab,kw OR ("decision support"):ti,ab,kw OR ("random forest"):ti,ab,kw OR ("support vector machine"):ti,ab,kw OR ("Xgboost"):ti,ab,kw OR ("adaboost"):ti,ab,kw OR ("gradient boosting machine"):ti,ab,kw OR ("regression tree"):ti,ab,kw OR ("least squares"):ti,ab,kw OR ("stepwise regression"):ti,ab,kw OR ("linear model"):ti,ab,kw OR ("logistic regression"):ti,ab,kw OR ("principle component analysis"):ti,ab,kw OR ("independent component analysis"):ti,ab,kw OR ("k means clustering"):ti,ab,kw |
| #5 | #3 OR #4 |
| #6 | ("COVID-19"):ti,ab,kw OR ("SARS-CoV-2"):ti,ab,kw |
| #7 | #1 AND #2 AND #5 NOT #6 |

[Web of Science](https://apps.webofknowledge.com/home.do?SID=6BQQjiiMCVa9MgFvRpC) (2975)

| Search | Query |
| --- | --- |
| #1 | TS=("severe") |
| #2 | ((TS=("pneumonia")) OR TS=("pulmonary inflammation")) OR TS=("pulmonary infection") |
| #3 | ((((((TS=("predict model")) OR TS=("risk prediction")) OR TS=("risk score")) OR TS=("prediction model")) OR TS=("prognostic model")) OR TS=("risk factor")) OR TS=("nomogram") |
| #4 | ((((((((((((((((((((((TS=("machine learning")) OR TS=("deep learning")) OR TS=("artificial intelligence")) OR TS=("neural network")) OR TS=("decision tree")) OR TS=("computational intelligence")) OR TS=("machine intelligence")) OR TS=("bayesian")) OR TS=("k-nearest neighbour")) OR TS=("decision support")) OR TS=("random forest")) OR TS=("support vector machine")) OR TS=("Xgboost")) OR TS=("adaboost")) OR TS=("gradient boosting machine")) OR TS=("regression tree")) OR TS=("least squares")) OR TS=("stepwise regression")) OR TS=("linear model")) OR TS=("logistic regression")) OR TS=("principle component analysis")) OR TS=("independent component analysis")) OR TS=("k means clustering") |
| #5 | #3 OR #4 |
| #6 | (TS=(COVID-19)) OR TS=(SARS-CoV-2) |
| #7 | #1 AND #2 AND #5 NOT #6 |
